# Transactions of the Gynæcological Society of Boston

**Published:** 1869-06

**Authors:** 


### TRANSACTIONS OF THE GYNÆCOLOGICAL SOCIETY OF BOSTON.

In accordance with the desire of several medical men of Boston and its vicinity, who had previously consulted upon the subject, a meeting was held on January 22d, 1869, for the purpose of establishing a Gynæcological Society, the first, so far as can be ascertained, of its kind in this country.

The meeting having been organized, Dr. Storer presented the arguments that had influenced the members to found the new Society. They were the following:—

1. That such a Society seems needed, in order to stimulate its members and the profession generally to a deeper sense of the importance of the diseases peculiar to women, and by the combination of individual effort to advance their knowledge of the causation, the pathology, and, still more, of the therapeutics of the lesions.

2. That it would do what can in no sense be just as well effected by other organizations already in existence. What is for everybody's interest is very apt to be done by no one. At a general medical and surgical society, there is not to be expected that intensity and focalization of scientific interest regarding special points which is necessary to advance the confines of a comparatively new science, which term Gynæcology must be confessed already to deserve.

3. That there can be no doubt that the special diseases of women comprise a vast variety of disturbances, direct and reflex, much of which is but partially understood or entirely unknown.

4. That these disturbances are of extreme importance, not merely to the individual sufferer, but with reference to her relations to her family and to society.

5. That their importance, their variety, and their frequency are but partially appreciated by the profession, and still less by the community.

6. That not merely is this statement true of great numbers of imperfectly educated physicians, but it is also true of many gentlemen of acknowledged skill as general practitioners, who have either lacked opportunity to perfect themselves in a knowledge of these diseases, or through an excessive conservatism have hesitated to acknowledge their existence.

7. That the marked advance of gynæcological science and art within the past twenty-five years, gives reasonable promise of a still more rapid progress in time to come.

8. That so far from its being a disgrace to a physician to be interested in uterine diseases, it should rather be considered, if he is known to have been thoroughly educated in general practice, an honor. As with the diseases of special sense, the eye and the ear for instance, the diseases of the throat and the chest, and of the mind, so here, all treatment must rest upon general principles;—and all methods of diagnosis, as all procedures of practice, not upon guesswork, but upon science and common sense.

9. That many of the great improvements that have been made have been American,—as the first successful performance of ovariotomy by McDowell; the suggestion of the rational treatment of vesico-vaginal fistula by Marion Sims; and of flexions of the uterus by Emmet;—American gynæcologists have already secured for the country a preëminent position in the world of science; it is for the members of this and kindred societies to make the position the more permanent.

10. And were there no other reason, the fact that every man owes to woman for her love in his infancy, in his childhood, and in his manhood, a debt that no devotion can ever repay; and when as physicians we reflect that her special diseases are manifold more in number, worse in severity, and more dangerous to physical and mental integrity than any affliction we ourselves are called to suffer, we should offer no less a sacrifice to the other sex than a life's work.

These arguments were commented upon approvingly by the gentlemen present, and it was furthermore decided,

11. That as the diseases of women are in great measure capable of being discovered and demonstrated, the same degree of disgrace should attach to physicians prescribing at random for married women complaining of pelvic symptoms, as to those who would do this in the case of diseases of the throat or eye, or who unjustifiably lengthen a patient's treatment for the sake of a larger fee.

12. That as in attending upon childbed all impurity of thought, and even the mental appreciation of a difference in sex are lost by the physician, and an imputation of them would be resented as an insult by the profession, so the care of uterine disease tends to inspire greater respect in a patient for her attendant, and in him for her. It is untrue to say that high-minded and delicate women instinctively desire to be attended by one of their own sex for these diseases, any more than in confinement, just as it is unquestionably the fact that because of the mental and physical disturbance temporarily induced even by healthy menstruation, women, the best of nurses, are unfitted to practise medicine and surgery, in any of their departments, with as much benefit to their patients or as successfully as men; and,

13. That as it is the duty of every searcher for truth to impart what he may find to his fellow-men, so it is incumbent upon the members of this Society to endeavor in every honorable way to exert an educative and persuasive influence upon the profession at large.

The constitution and by-laws were then adopted. They state the purpose of the society to be the advancement of gynæic science and art, and their due recognition, both in Boston and throughout the country; and recognize as the code of ethics that of the American Medical Association.

---

Dr. H. B. Storer presented to the Society a masked patient concerning whom he desired advice, the case being one of

OBSTINATE EROTOMANIA.

The history was as follows:—

Age of the patient, 50; American, unmarried, and from the country. Climacteric passed several years since; previous to which time, and subsequently, the general health has been good. At twenty-five, coitus was once indulged in with the overseer of a mill, at which many foreigners were employed; and upon the remembrance of this the patient has lived. The mental and physical condition are both peculiar. There is action and reason—and the question is to decide whether the brain here chiefly affects the genitals, the genitals the brain, or each the other. There has for many years existed a troublesome pruritus, and a constant twitching of the clitoridal region, analogous, apparently, to that of the infra-orbital muscles occasionally noticed. These have been attended by an inordinate longing for the other sex, and a frequent indulgence in masturbation. In addition to these appetites, under the circumstances not at all unusual, there exists a remarkable delusion. The patient thinks that the knowledge of her fault, committed so many years ago, has been communicated backwards and forwards among the Irish throughout the country, so that every man or woman of that nation whom she meets seems by word or by deed to be taunting her. If she hears an Irishman say to his comrade, "It's very hot to-day," she imagines that he applies the expression to her; if he says that "It's very cold," he is upbraiding her for an indifference that she endeavors in vain to attain. So that every person of the kind whom she meets starts, through her morbid self-consciousness and remorse, the old disordered train of ideas, and these, reflexly and always, kindle the vulval congestion, which almost inevitably culminates in orgasm.

Before the patient consulted Dr. S., her clitoris had been excised at Chicopee; no benefit being obtained. After the employment of every local sedative he could think of, borax, tobacco, morphia in lotion and by hypodermic injection, hydrocyanic acid, acetate of lead, the vapor of chloroform, etc., etc., and a corresponding appeal to antiphrodisiacs, exhibited by the mouth, as bromide of potassium up to an hundred-grain doses, etc., etc., without avail, Dr. Storer quieted the pruritus by superficial vesication with a saturated aqueous solution of carbolic acid. The muscular twitching still remained. There was no clitoris left to excise, even if Dr. S. had believed in the efficacy of Mr. Baker Brown's treatment, which, from its unsuccessful employment at his hands in other cases, he did not. He had resorted to an operation which might be a novel one: by passing, with a curved needle, ligatures beneath the crura clitoridis, and down against the pubic arch, at a distance from each other of nearly half an inch, and allowing these to slough out, he had divided, so far as seemed possible, all nervous communication with the affected part. Relief, however, had been but partial. The actual cautery and cantharidal collodion had each given temporary quiet, but the symptoms returned. The vagina, urethra, and bladder had been carefully examined, but nothing abnormal could be found. The uterus seemed perfectly healthy, as small and supple as in a virgin who had passed the climacteric, and not at all displaced. To make assurance doubly sure, and to get, if possible, a reflex effect, the acid nitrate of mercury was applied without and within the uterine cervix. No clitoridal response of any kind was elicited.

The rectum was searched for ascarides—none were found—some small hemorrhoids were excised, and the sphincter ani ruptured by forcible dilatation, but the twitching continued as badly as ever. The liver was appealed to in vain, and in vain had blisters been put behind the ears. In desperation, Dr. S. had jokingly said to the patient he believed he should have to sew up her vulva closely, and, now, here was the woman daily begging him to do so, or end her misery by putting an end to her existence. He had little doubt, from the history of the case, that the mental disturbance was in part, at any rate, of pelvic causation, however much the local irritation existing at present was dependent upon the former; and he had little faith that the ordinary moral treatment relied upon in insane asylums for female patients would do this woman any good. He had not as yet iced the spine; and was about inserting a seton in the nucha. He was loath to throw the case aside, if there were any reasonable ground of treatment remaining to be tried; he therefore appealed to the society for aid.

Dr. Wheeler, of Chelsea, after carefully examining the case, remarked, that it was certainly a very unusual and interesting one. He had no doubt in his own mind that in very many instances of insanity in women a cure was possible, and could only be obtained by local treatment. In such a case as that now presented, this must necessarily be often empirical; yet, under the circumstances, such was both justifiable and advisable, and should be long persisted in.

Drs. Warner, Bixby, and Dutton had each seen the case with Dr. Storer, and had studied many details of the treatment.

Dr. Field, of Newton Corners, stated that here we had an instance of the conflict so often observed by physicians between what is demanded by deference to public morality, and what seems required for a patient's health. If this woman could go masked as she is at the present moment to a house of prostitution, and spend every night for a fortnight at sexual hard labor, it might prove her salvation; such a course, however, the physician cannot advise. And so with masturbation. In a case like the present, its indulgence may be a means of getting temporary relief from a local fret, whose influence upon the mind, if not thus relieved, might prove more disastrous.

Dr. Sharp suggested the employment of galvanism, especially by faradization, and of an appeal, in succession, to the various regions of the spinal cord. These had not as yet been resorted to; it was possible their use might yet solve the problem.

The Society then adjourned.—*American Jour. of Obstetrics.*

---

MEDICAL VACANCIES IN THE REGULAR ARMY.—At the date of the Surgeon-General's report (October 20th, 1868), there were forty-nine vacancies in the grade of assistant-surgeon.—*The Medical Record.*

